# Minimal graft site morbidity using autogenous semitendinosus graft from the uninjured leg: a randomised controlled trial

**DOI:** 10.1007/s00167-021-06686-6

**Published:** 2021-08-08

**Authors:** Christoffer von Essen, Sebastian McCallum, Karl Eriksson, Björn Barenius

**Affiliations:** 1 Department of Orthopaedics, Stockholm South Hospital, Karolinska Institutet, Stockholm, Sweden; 2 Capio Artro Clinic, FIFA Medical Centre of Excellence, Sophiahemmet Hospital, Valhallavägen 91, 11486 Stockholm, Sweden

**Keywords:** ACL, ACL reconstruction, Contralateral, Semitendinosus, Isokinetic

## Abstract

**Purpose:**

To quantify the effect on strength of semitendinosus (ST) graft harvest by comparing isokinetic and isometric muscle strength.

**Methods:**

A cohort of 140 patients underwent anterior cruciate ligament (ACL) reconstruction (ACLR) and were randomized to ipsilateral or contralateral ST graft harvest. Isokinetic and isometric muscle strength testing using a dynamometer were collected for the operated and non-operated leg. Patients were assessed pre-surgery and at 6, 12 and 24 months after reconstruction.

**Results:**

ST graft harvest reduced isokinetic flexion muscle strength for 6 months. At 12 months follow up there was no significant difference between the two groups and they were all stronger than pre-injury. No other significant differences were found in any primary or secondary outcome measurements.

**Conclusion:**

Solitary ST graft harvest does not appear to result in a permanent reduced isometric or isokinetic quadriceps muscle strength on the side where the graft is harvested. A reduction in hamstring muscle strength of less than 10% can be seen at short-term follow-up with full recovery by 12 months. Most patients report little or no donor site pain. Given these findings, ST autograft is an alternative graft choice that could be used for various reconstructions in terms of donor site morbidity.

**Level of evidence:**

Level II.

## Introduction

Anterior cruciate ligament (ACL) reconstruction (ACLR) can be performed using an allograft or autograft, including the patellar tendon (BPTB), hamstring tendon (HT), and quadriceps tendon (QT), and the ultimate choice of graft is made considering the pros and cons of each graft type with respect to the surgeon’s and patient’s preferences.

While each graft has relative benefits and drawbacks, there is no evidence demonstrating that one has clear superiority over the others, although HT grafts are considered to create less donor site morbidity than BPTB [6, 17, 19, 23, 24].

HT is the most commonly used autograft for ACLR [1, 4, 5]. Further, HT may also be used as an alternative in both foot and shoulder surgeries[13, 16]. HT harvest has been extensively studied together with ACLR and both the semitendinosus (ST) and gracilis (G) tendons have been shown to regenerate to a certain extent after harvest, although functional deficit, especially decreased hamstring strength, may persist [3, 8, 11, 20]. Studies have indicated that harvesting only ST (and not both ST and G) is associated with an improved restoration of both isokinetic and isometric hamstring strength, consequently, it has been recommended to preserve the G [7, 12, 18].

Few studies have examined the effects of solitary HT harvest. Yasuda et al. [25] compared ipsilateral (IL) and contralateral (CL) ST-G tendon harvest, but did not find any significant effect on quadriceps muscle strength, although hamstring muscle strength was reduced up to one year after surgery. Paterson et al. [15] conducted strength testing on 26 patients who used HT graft for ankle reconstruction and found no difference between the nonoperated and the operated leg. To our knowledge, there have been no studies examining the morbidity caused by harvesting a solitary ST graft independent of the morbidity associated with the ACLR itself. This study aimed to determine the effect of ST graft harvest by comparing isokinetic and isometric muscle strength between the nonoperated (NO) and operated (O) leg, in patients undergoing ACLR performed with either IL or CL ST-graft harvest.

## Materials and methods

Approval for the study was obtained by the regional ethics committee at the Karolinska Institute, Stockholm Sweden (reference no. 2013/1398-31/2).

**Fig. 1:**
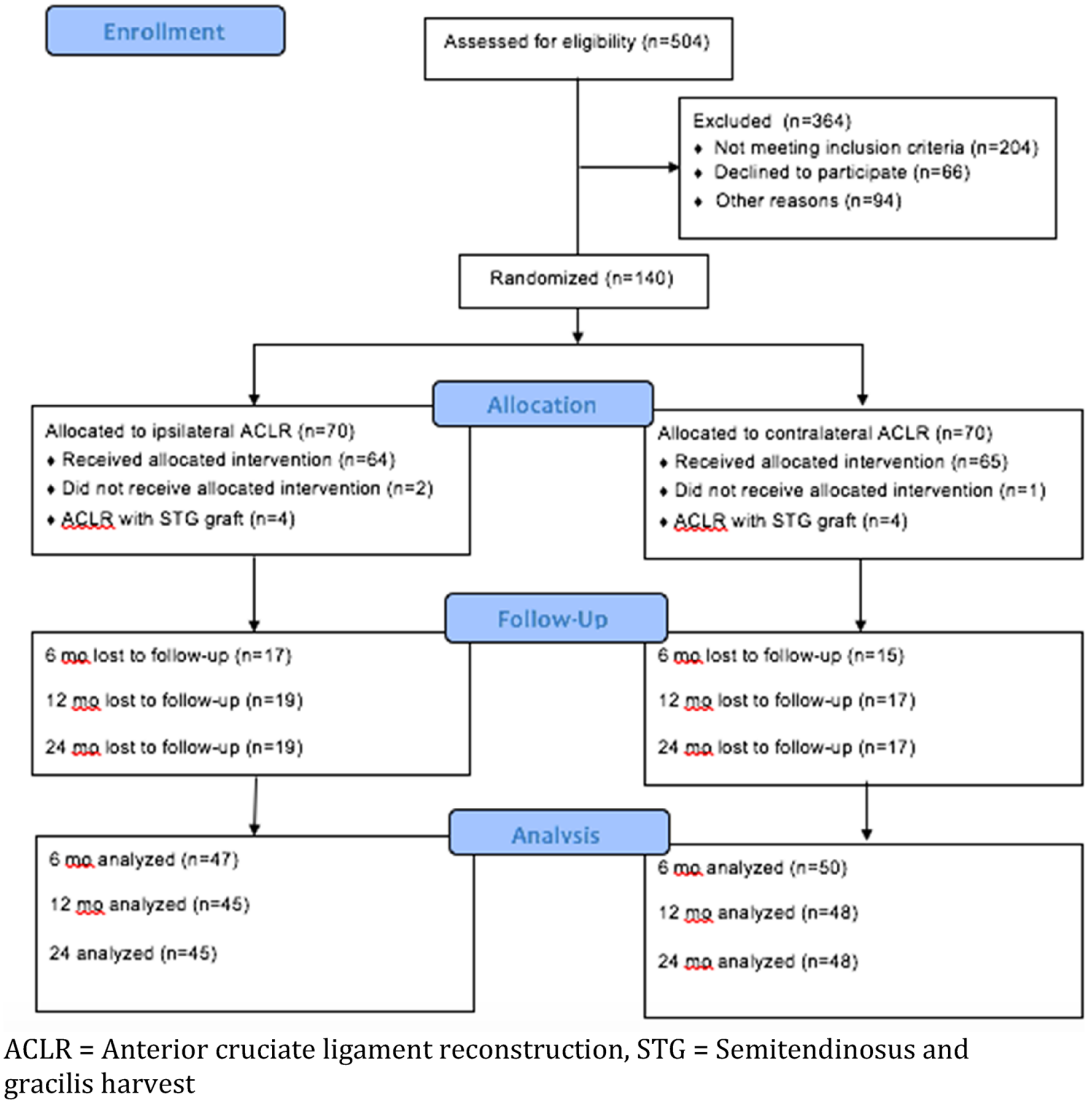
Flow diagram of patients’ progress through the phases of the study. *ACLR* anterior cruciate ligament reconstruction, *STG* semitendinosus and gracilis harvest

From 2013 to 2017, all patients with a verified ACL injury at the orthopedic clinic were screened for inclusion. Eligibility was determined using defined study exclusion and inclusion criteria, listed in Table 1. Out of 504 patients, 140 patients were included (Fig. 1). Prior to participation, each eligible patient received standardized information about the trial. Randomisation was performed with the sealed envelope system in batches of 20. Patient demographics are reported in Table 2, with no significant differences between the groups. Patients were assessed preoperatively and postoperatively at 6, 12 and 24 months.

**Table 1:** Inclusion and exclusion criteria

Inclusion criteria	Exclusion criteria
Unilateral ACL injury	Contralateral ACL injury
Age 18–50 years	PCL injury
	LCL injury
	MCL injury ≥ grade 2
	Multiligament injuries
	Significant hamstring injury

*ACL* anterior cruciate ligament, *PCL* Posterior cruciate ligament, *LCL* Lateral collateral ligament, *MCL* Medial collateral ligament

**Table 2:** Baseline characteristics of the study population

	Total (*n *= 129)	Ipsilateral ACLR *n *= 64	Contralateral ACLR *n *= 65	*p* value
Age at inclusion, mean ± SD	33 ± 9	33 ± 9	31 ± 9	n.s
Gender: male, *n* (%)	75 (58)	33 (52)	42 (65)	n.s
BMI mean ± SD	25 ± 3	25 ± 4	24 ± 3	n.s
Participating in sport when injured *n* (%)	91 (71)	49 (77)	41 (63)	n.s
Time injury-recon median months (range)		6(1–188)	5 (1–250)	n.s
Additional injury *n* (%)	75 (58)	37 (58)	38 (59)	n.s
Medial meniscus *n* (%)	52 (40)	27 (42)	25 (39)	n.s
Lateral meniscus *n* (%)	27 (21)	16 (25)	11 (17)	n.s
Medial repair *n* (%)	20 (16)	10 (16)	10 (15)	n.s
Lateral repair *n* (%)	13 (10)	6 (9)	7 (11)	n.s
Cartilage inj *n* (%)	19 (15)	6 (9)	13 (20)	n.s

Demographic Data at baseline, displayed as mean ± SD, number and percentage, respectively

*ACLR* anterior cruciate ligament reconstruction

### Surgical technique and post-operative treatment

Standardized arthroscopic anatomcial ACLR was performed using a quadruple ST-graft. All ACLRs were done by two experienced surgeons. The ST grafts were harvested through an anteromedial longitudinal incision and bluntly dissected with a tendon stripper.

All patients were instructed to participate in a standardized post-surgical rehabilitation plan. Patients were allowed full weight-bearing immediately. Competitive sports were not permitted within 9 months after surgery. There are several rehabilitation centers affiliated with the study hospital, familiar with our rehabilitation plan, however, patients were allowed to choose other sites for rehabilitation if they wished.

### Patient evaluation

Demographic data were obtained at baseline. Isokinetic and isometric strength testing of both extension and flexion was measured using the Biodex System 3 (Biodex Medical Systems, Shirley, NY, USA). Each leg was tested for isokinetic peak torque at 60°/s and180°/s, for isometric torque strength at 60°, and Total work 180°. The tests were conducted preoperatively and 6, 12 and 24 months postoperatively. A comparison was made to measurements of the non-injured leg, recorded prior to surgery. As a measurement of functional strength, the quadriceps muscle was measured approximately 10 cm above the patella to evaluate atrophy. A direct question regarding donor site soreness was also asked at the given time points according to the International Knee Documentation Committee (IKDC) ligament standard evaluation form [10], grading it between none, mild, moderate and severe. Functional scores, IKDC 2000 and Lysholm score [22], were also obtained.

### Statistical analysis

Statistical analysis was performed with the SPSS (version 25.0, IBM Corp., NY, USA) software package. To compare parametric and nonparametric variables between the groups the independent t-test and Mann–Whitney *U* test were used. Nominal variables were tested by Fisher’s exact test. Paired-sampled *t* test was used for Longitudinal statistics for normally distributed scale variables. *p* values were considered significant at *p* < 0.05.

The sample size calculation for the study was originally designed to compare isometric hamstring strength at 6 months between patients who underwent ACLR using an ST graft from either the ipsilateral or contralateral leg. To obtain an 80% power, a sample size of 74 patients was needed.

To determine the effect size and the power of the study, a post hoc power analysis using G*Power 3.1.9.2 (Franz Paul, Kiel, Germany) was used. The analysis revealed, based on isokinetic hamstring strength at the velocity of 180°/s at 6 months, that a sample size of 70 patients in each group would yield a power of 98% to detect a mean of 10% difference in muscle strength and an effect size of 0.81 was obtained.

## Results

### Muscle strength according to Biodex^®^

For isokinetic flexion muscle strength, the CL group was significantly weaker at 60°/s (*p *= 0.001) and180°/s (*p *= 0.001) at 6 months compared to baseline. As early as 12 months there were no significant differences between the groups and they were all stronger than pre-injury (Table 3). This was also true for Total Work (Table 3). The CL group was also always stronger than 90% of baseline values at all measured time points. Isokinetic extension muscle strength had improved in both groups at all velocities at all measured time points with no difference in strength between them (Table 3).

**Table 3:** Result of isokinetic torque testing at different speeds and total work at 180°/s isokinetic

	60°/s			180°/s		
	IL group	CL group	*p* value	IL group	CL group	*p* value
Flexion torque						
6 m (*n* = 47/50)	111.5 ± 18.2	91.4 ± 25.4	0.001	113.1 ± 17.2	92.8 ± 27.7	0.001
12 m (*n* = 44/48)	107.5 ± 21.5	96.6 ± 25.5	n.s	110.7 ± 19.6	98.7 ± 28.1	n.s
24 m (*n* = 44/47)	111.6 ± 19.8	105.3 ± 21.8	n.s	115.2 ± 16.2	109.2 ± 19.6	n.s
Extension torque						
6 m (*n* = 47/50)	105.9 ± 16.4	102.3 ± 22.8	n.s	109.5 ± 14.3	102.8 ± 20.2	n.s
12 m (*n* = 44/48)	100.8 ± 25.5	103.8 ± 21.6	n.s	106.7 ± 20.1	105.6 ± 19.2	n.s
24 m (*n* = 44/47)	108.1 ± 15.1	104.4 ± 20.4	n.s	112.1 ± 12.8	109.4 ± 14.4	n.s
The average total work at 180°/s						
6 m (*n* = 47/50)	113.7 ± 23.8	88.0 ± 26.4	0.005	107.5 ± 17.1	101.6 ± 26.3	n.s
12 m (*n* = 44/48)	110.8 ± 26.0	98.5 ± 31.1	n.s	104.2 ± 21.9	105.0 ± 19.7	n.s
24 m (*n* = 44/47)	116.8 ± 21.1	107.6 ± 24.7	n.s	111.2 ± 16.6	108.8 ± 15.7	n.s

The nonoperated leg in the IL group was stronger for isometric muscle strength than pre-injury and continued to be so during the study. Despite this, there were no significant differences, neither in flexion or extension, at any time point (Table 4).

**Table 4:** Result of isometric torque at 60°

	Flexion			Extension		
	IL group	CL group	*p* value	IL group	CL group	*p* value
Isometric torque at 60°						
6 m (*n* = 47/50)	109.8 ± 18.3	102.3 ± 25.6	n.s	100.4 ± 18.4	94.3 ± 23.2	n.s
12 m (*n* = 44/48)	110.1 ± 22.4	108.4 ± 27.7	n.s	104.2 ± 24.8	98.0 ± 24.4	n.s
24 m (*n* = 44/47)	112.5 ± 20.7	110.8 ± 25.8	n.s	107.8 ± 18.7	107.1 ± 25.2	n.s

The average isometric torque muscle strength at 6o degrees and total work at 180°/s isokinetic across time by limb (based on intervention) displayed as a mean percentage with reference pre-uninjured leg set at 100

IL = ipsilateral hamstring graft, i.e. the leg without any surgery CL = contralateral hamstring graft, i.e. the leg with semitendinosus harvest

There were no differences in muscle hypotrophy between the groups (Table 5). Further, most study participants did not have any donor site pain (66%), and only three patients (6%) suffered mild pain from the donor site and none reported severe pain (Table 6).

**Table 5:** Functional strength

	Ipsilateralt ACLR	Contralateralt ACLR	*p* value
Thigh circ. 10 cm above patella in cm (SD)			
6 m *n* = 51/53	48 (5)	49 (4)	n.s
12 m *n* = 46/52	49 (4)	49 (3)	n.s
24 m *n* = 48/50	49 (5)	50 (4)	n.s

*ACLR* anterior cruciate ligament reconstruction

**Table 6:** Donor site soreness for contralateral ACLR

	None	Mild pain	Moderate pain	Severe pain
6 m *n* (%)	34 (67)	17 (33)	0	0
12 m *n* (%)	37 (73)	13 (25)	1 (2)	0
24 m *n* (%)	31 (66)	13 (28)	3 (6)	0

Objective score ranging from none to severe

*ACLR* Anterior cruciate ligament reconstruction

### Patient-related outcome

**Fig. 2:**
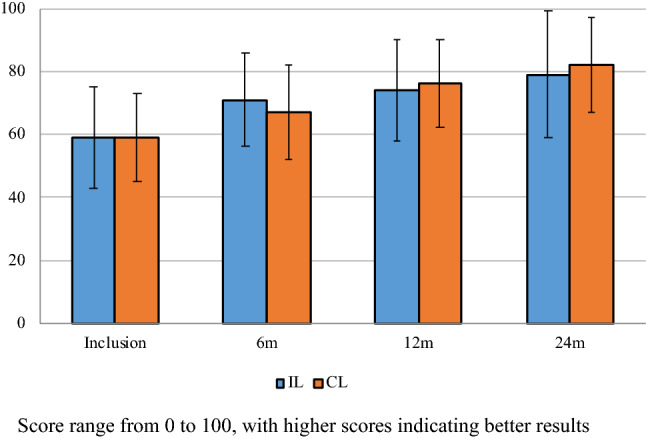
Lysholm score. Score range from 0 to 100, with higher scores indicating better results

**Fig. 3:**
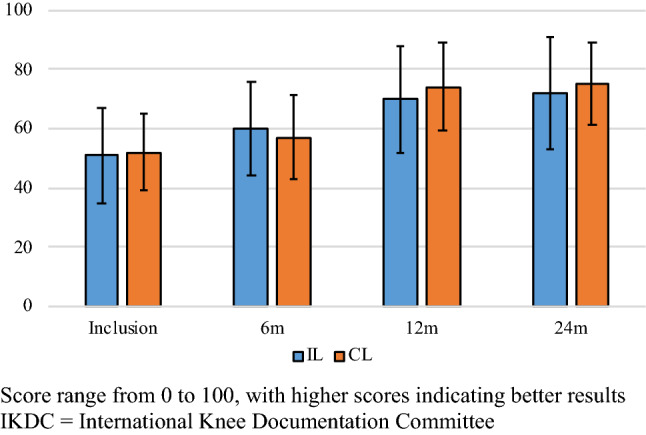
IKDC subjective knee score. Score range from 0 to 100, with higher scores indicating better results. *IKDC* International Knee Documentation Committee

The IKDC and Lysholm scores over time are presented in Figs. 2 and 3 respectively. There were no significant differences between the groups at any time point. Scores significantly increased from preoperatively to 24 months.

## Discussion

The most important finding of this study was a demonstrable deficit in hamstring strength at 6 months that can be attributed specifically to ST tendon grafting, not the ACL injury itself. To our knowledge, no other study has demonstrated and quantified the morbidity after an isolated ST tendon graft harvest, with regards to postoperative quadriceps and hamstring muscles strength, without the influence of an additional ACLR in the same knee. This is important knowledge for the surgeon when choosing an ST autograft, both in ACLR but also when using an ST graft in various other surgeries.

In the limbs that were uninjured but used for graft harvesting, hamstring strength was significantly reduced for 6 months, however, quadriceps strength was not adversely affected. This reflects the findings of Yasuda et al. [25]. In their study, ST-G harvest did not have any significant effect on quadriceps muscle strength but hamstring strength was significantly reduced for more than 12 months. Yasuda reported a 20% deficit in isometric hamstring strength at 6 months follow up after graft harvest, while in this study there was a 6% deficit in isometric hamstring strength found at the same time point. A potential explanation for this difference could be the preservation of the gracilis tendon in our study, with gracilis harvest likely resulting in an increased effect on isokinetic hamstring strength. Sharma et al. [18] noted similar trends, although their autografts were taken from the ipsilateral leg. The effect of an isolated ST graft harvest on hamstring strength seems to be minor in comparison to the effect from a combined ACLR and complete hamstring tendons harvest. From this study, it appears that strength deficits following isolated ST graft harvest are recovered at 2 year follow up, with no persisting long-term morbidity. This contrasts with the findings of Nakamura et al. [14] and Aune et al. [2], who reported a 10 to 20% deficit in hamstring strength two years postoperatively. One explanation for this discrepancy could be the fact that ACL surgery was performed in the same leg in these studies, affecting rehabilitation of the hamstring muscle.

It has been shown that following graft harvest the hamstring tendon regenerates to form a tendon-like structure [8, 9, 12]. A systematic review by Suijkerbuijk et al. [21] found that the ST regeneration-rate was almost 80 percent within a year, though this study did not assess muscle strength or function. Although a limb that has undergone an isolated ST harvest does not recover strength to a preoperative reference level within 12 months, patients retain the ability to produce isokinetic peak flexion torques over 90% of reference levels at all velocities as early as 6 months post-surgery. It is likely that the recovery after an isolated ST harvest is quicker due to the lesser surgical trauma facilitating more effective rehabilitation.

The strengths of this study are the randomized design and the same standardized surgical technique. All surgeries were performed by two experienced orthopedic surgeons. The patients were comparable in demographic data for the two groups, so differences found should be attributable to the different surgical scenarios. The large sample size is another strength of this study. There are limitations to the study. Firstly, the pre-study power analysis was only made to detect differences in hamstring strength in the injured leg. However, a post hoc power analysis yielded an effect size of 0.81 and a 98% power detect differences regarding isokinetic hamstring strength. Second, a large number of patients were lost to follow-up. A further limitation of the study was that the patients could choose where they undertook rehabilitation. As many patients partake in a non-surgical treatment period before surgical treatment is chosen, many elected to continue rehabilitation at the same center where contact was already established. As such, there may have been some variation in the exact rehabilitation provided which may be a confounding factor. Conversely, results should therefore better reflect a clinical setting and be more generalizable.

### Conclusion

Solitary ST graft harvest does not appear to result in a permanent reduced isometric or isokinetic quadriceps muscle strength on the side where the graft is harvested. A reduction in hamstring muscle strength of less than 10% can be seen at short-term follow-up with full recovery by 12 months. Most patients report little or no donor sight pain. Given these findings, ST autograft is an alternative graft choice that could be used for various reconstructions in terms of donor site morbidity,
